# SizeExtractR: A workflow for rapid reproducible extraction of object size metrics from scaled images

**DOI:** 10.1002/ece3.8724

**Published:** 2022-03-14

**Authors:** Liam Lachs, Fiona Chong, Maria Beger, Holly K. East, James R. Guest, Brigitte Sommer

**Affiliations:** ^1^ 5994 School of Natural & Environmental Sciences Newcastle University Newcastle upon Tyne UK; ^2^ 4019 Department of Biological & Marine Sciences University of Hull Hull UK; ^3^ 4019 Energy and Environment Institute University of Hull Hull UK; ^4^ School of Biology Faculty of Biological Sciences University of Leeds Leeds UK; ^5^ Centre for Biodiversity Conservation Science School of Biological Sciences The University of Queensland Brisbane Queensland Australia; ^6^ 5995 Department of Geography and Environmental Sciences Northumbria University Newcastle upon Tyne UK; ^7^ 1994 School of Life Sciences University of Technology Sydney Sydney New South Wales Australia; ^8^ 1994 School of Life and Environmental Sciences The University of Sydney Sydney New South Wales Australia

**Keywords:** coral reefs, image analysis, object dimensions, population dynamics, reproducibility, size frequency distributions, size metrics, time saving

## Abstract

1. Size is a biological characteristic that drives ecological processes from microscopic to geographic spatial scales, influencing cellular energetics, species fitness, population dynamics, and ecological interactions. Methods to measure size from images (e.g., proxies of body size, leaf area, and cell area) occur along a gradient from manual approaches to fully automated technologies (e.g., machine learning). These methods differ in terms of time investment, expertise required, and data or resource availability. While manual methods can improve accuracy through human recognition, they can be labor intensive, highlighting the need for semi‐automated, and user‐friendly software or workflows to increase the efficiency of manual techniques.
2. Here, we present SizeExtractR, an open‐source workflow that enables faster extraction of size metrics from scaled images (e.g., each image includes a ruler) using semi‐automated protocols. It comprises a set of ImageJ macros to speed up size extraction and annotation, and an R‐package for the quality control of annotations, data collation, calibration, and visualization.
3. SizeExtractR extracts seven common size dimensions, including planar area, min/max diameter, and perimeter. Users can record additional categorical variables relating to their own study, for example species ID, by simply adding alphanumeric annotations to individual objects when prompted. Using a population size structure case study for hard corals as an example, we show how SizeExtractR was used to quantify the impact of mass coral bleaching on coral population dynamics. Lastly, the time saving benefit of using SizeExtractR was quantified during a series of timed image analyses, revealing up to a 49% reduction in image analysis time compared to a fully manual approach.
4. SizeExtractR automatically archives results, allowing re‐analysis of size extraction and promoting quality control and reproducibility. It has already been employed in marine and terrestrial sciences to assess population dynamics and demography, energy investment in eggs, and growth of nursery reared corals, with potential to be applied to a wide range of other research fields.

## Graphical abstract

Size as a biological characteristic is fundamental to ecology and evolution. SizeExtractR is a new software and workflow for extracting size metrics from scaled images, reducing analysis time by almost half, archiving all relevant data files, and integrating quality control checks into the workflow. This new software solution has potential to be used across disciplines in ecology and environmental sciences.

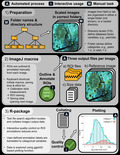

## 1 INTRODUCTION

As a biological feature, size has a fundamental influence on the ecology and evolution of all organisms (Tan et al., 2021), yet our ability to quantify size rapidly, consistently, and accurately from images across disciplines remains limited (Edmunds & Riegl, 2020; Weinstein, 2018). The importance of size extends to all scales of biological and ecological organization: cell size can indicate resource availability (Paxton et al., 2016); organ/body‐part size can be used as a proxy for somatic and reproductive investment (Stevens et al., 2000); and body size can influence fitness and competitive success (Dickerson et al., 2002; White et al., 2018). Quantification of size can elucidate vital rates, such as recruitment, growth, reproduction, and senescence (Cant et al., 2020) and can reveal the size structure of species populations (Lachs et al., 2021), which ultimately define population proliferation or demise. As such, size is the focus of a vast literature on ecological theory, such as the Island Rule (increasing body size with island size), and is a central component of contemporary ecological and demographic research (Edmunds & Riegl, 2020). We consider ‘size’ as an umbrella term for numerous ecologically meaningful measurements (e.g., proxies of body size, leaf area, fish/shell length, cell area, or maximum and minimum diameters). There is a growing reliance on measuring size from scaled imagery (i.e., images containing objects of known length) (Beaudouin et al., 2015; Benton et al., 2008; Precoda et al., 2018). This has improved sampling efficiency greatly (Lachs et al., 2021; Sommer et al., 2014), and highlights the growing demand for reliable, user‐friendly software or workflows to rapidly quantify size from scaled images.

While manual approaches to extract size from images are commonly used in science (Weinstein, 2018), automated image analysis technologies are undergoing rapid advancements (Hagendorff & Wezel, 2020). For example, in ecology, machine learning technologies can automatically measure object sizes (size of any irregular 2D region in an image) from vast image datasets, given enough training data and appropriate standardization of images (Alonso et al., 2019; Kloster et al., 2014; Monkman et al., 2019; Wäldchen & Mäder, 2018; Weinstein, 2018). However, for some research projects, such techniques are not applicable and better returns‐on‐investment can be achieved from using manual methods (e.g., for small image datasets, or low quality or high complexity images). At the center of the manual–automatic gradient, generic particle analyzers, such as the BioVoxxel Toolbox (Brocher, 2014), can be used to batch process particle size extraction. However, such approaches are often designed for standardized microscopy images, and are not appropriate for complex ecological imagery with chaotic backgrounds (e.g., forest floor). The advantage of fully manual image analysis methods is rooted in the accuracy of human recognition in tasks like species identification, boundary delineation, and the ability to record ad hoc observations (e.g., health status) without having to hard‐code them into identification algorithms. Human recognition and manual extraction of size measures have proven fundamental to building size datasets for ecology research. For example, manual extraction of size metrics has recently been used in assessments of population dynamics for marine and terrestrial fauna (Beaudouin et al., 2015; Benton et al., 2008; Bogdan et al., 2021), estimation of size‐dependent disease susceptibility (Bruno et al., 2011), measurement of growth to run integral projection models (Cant et al., 2020; Precoda et al., 2018), and testing the inter‐generational effects on reproductive effort (Plaistow et al., 2006).

Manual image analysis methods (e.g., size extraction, or measurement of RGB color as red, green, and blue), such as those conducted using ImageJ (Schneider et al., 2012), typically require protocols that can be slow, labor‐intensive, and prone to human error in data handling. Thus, there is an urgent need for robust software or workflows that focus researchers’ efforts on complex tasks that require human recognition, while automating monotonous tasks that are easily programmed (e.g., exporting results to spreadsheets). Such semi‐automated image annotation options would improve scientific reproducibility and support ecologists and biologists when machine learning methods are unsuitable. To address these problems and gaps, we present a semi‐automated, free‐to‐use image analysis workflow called SizeExtractR, built using ImageJ and R.

## 2 SizeExtractR GETTING STARTED

**FIGURE 1:**
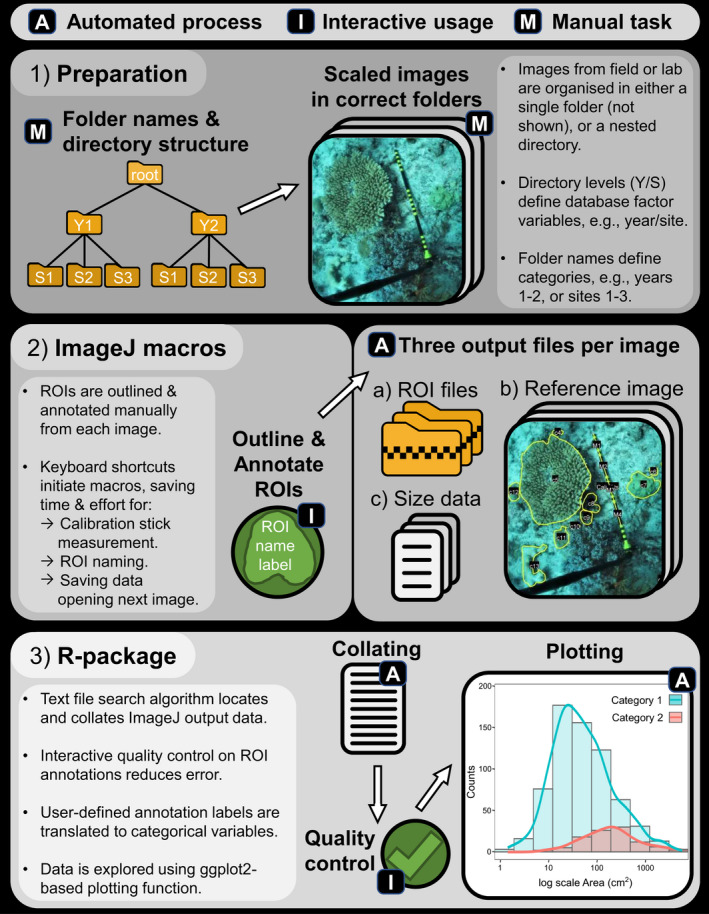
Conceptual diagram of the SizeExtractR workflow, highlighting the automated (A), interactive (I, green), and manual (M) steps. (1) *Preparation*: Images from field or laboratory work that each include a scale of known length (e.g., 10cm banded stick), are either put in a single folder (not shown) or organized among multiple folders with a nested directory structure that will later define optional categorical database variables (e.g., Year subfolders [categories: 1, 2], within Site subfolders [categories: 1, 2, 3], within a root folder). (2) *ImageJ macros*: The images are annotated using SizeExtractR‐macros and default ImageJ tools (e.g., freehand tool) to outline all regions of Interest (ROIs) and label them according to a user‐defined labeling system (e.g., to record user‐defined categorical variables, see Figure 2). Three output files are produced per image: a text file containing uncalibrated ROI size measurements and alphanumeric ROI annotations; a zip folder containing ROI files; and a reference image showing all ROI outlines and annotations. (3) *R*‐*package*: SizeExtractR‐package is then used to (a) conduct quality control and check ROI annotations; (b) calibrate size measurements, extract user‐defined categorical variables from folder names and ROI annotation labels and collate all data; and (c) plot size frequency distributions among categorical grouping variables

SizeExtractR is an open source workflow that enables fast extraction of object sizes from scaled images (i.e., images containing a size reference scale), combining the accuracy of human recognition with the speed of semi‐automated protocols. The SizeExtractR workflow is completed using both ImageJ and R, but no prior knowledge of either software is a prerequisite for usage. A full methodology including installation instructions, a step‐by‐step guide, and a worked example are provided in the Supplementary User Guide. First, images should be manually organized by the user either within a single folder, or in a directory tree where folders relate to some consistent categorical hierarchy (e.g., site‐folders within year‐folders). Second, a set of custom ImageJ macros (referred to as SizeExtractR‐macros, programs that automate processes) are used to facilitate manual outlining and annotation of objects and saving of size data and reference data files (Figure 1). These semi‐automated macros are initiated using keyboard shortcuts and prompt the user for input where necessary (i.e., outlining and annotating). By removing the need for users to search through drop‐down menus to set tools in ImageJ and navigate pop‐up boxes to save output files, this workflow saves considerable time. Users manually outline regions of interest (ROIs) with the freehand tool using a mouse or touchpad hardware. Importantly, each image must include a scale of known length (e.g., ruler) for calibration later. Finally, an R‐package (referred to as the SizeExtractR‐package, see supplementary user guide for install instructions) is used to check for human errors made during image annotation, perform size calibrations, collate all data to build a single size dataset, and plot size frequency distributions (Figure 1). Together, the SizeExtractR ImageJ macros and R package considerably improves transparency and traceability (in terms of fully documenting work), and image analysis time, whilst reducing the chance of human error (specifically in allocating the correct user‐defined categorical variables and naming of data files).

### 2.1 ImageJ‐macros

**FIGURE 2:**
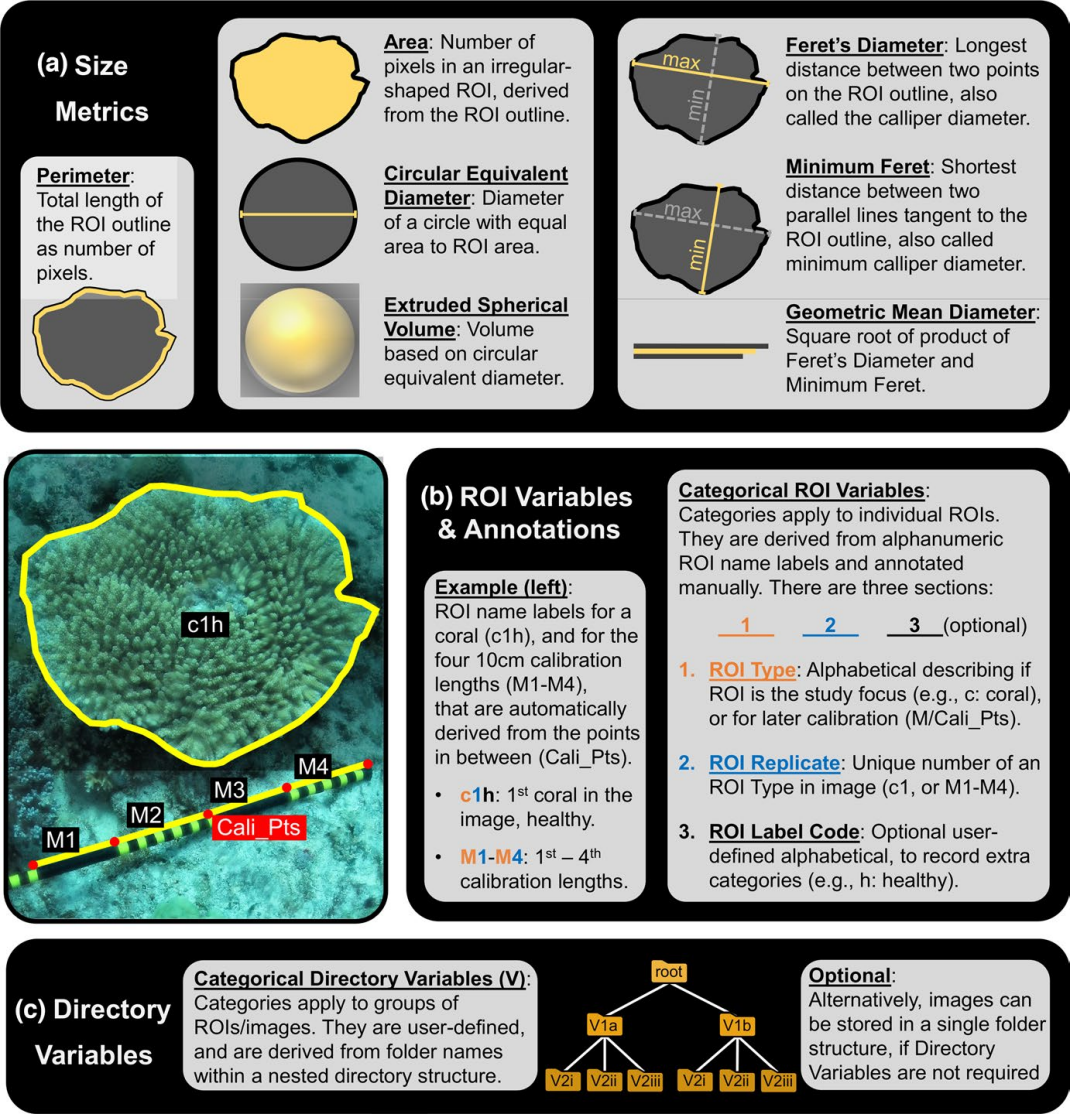
Illustration showing the variables that can be measured and recorded using SizeExtractR. (a) Seven size metrics are automatically measured with SizeExtractR‐macros, once ROIs are fully annotated. (b) An example annotation shows how categorical ROI Variables are recorded based a simple labeling system. (c) Optional categorical Directory Variables can also be included in analyses to record additional notes on each ROI and are derived from folder names

The SizeExtractR‐macros are designed to improve the reproducibility and speed of manually outlining and annotating objects to measure their size and other categorical features (e.g., species ID). SizeExtractR‐macros measure seven common size metrics that describe the dimensions of irregular‐shaped objects (saved in ImageJ as ROIs): area, circular equivalent diameter, extruded spherical volume, max/min Feret's diameter, geometric mean diameter, and perimeter length (Figure 2). Scale length is also recorded (e.g., of a ruler), and is used later in R to calibrate the size measurements from pixels to case‐specific units (e.g., cm and cm^2^).

SizeExtractR is also used to facilitate the recording of user‐defined categorical variables that relate to individual ROIs (e.g., species identity or health status category), referred to as ‘ROI Variables’. After outlining a ROI, an automated prompt requests users to manually enter an alphanumeric annotation, or ‘ROI name label’. This label is composed of three sections (Figure 2): Section 1—the alphabetical ROI Type, which either classifies objects of interest (e.g., the study species) or measurement scales in each image (e.g., a ruler); Section 2—the numerical ROI Replicate, which is a unique number given to each ROI within a specific image (see M1‐M4 in Figure 2), facilitating post‐hoc quality control and ROI re‐analysis; Section 3—the alphabetical ROI Label Code, which is optional and can be used to record any additional notes relating to specific ROIs (e.g., a damaged sample, or different morphology).

In contrast to categorical ROI Variables which differ per ROI, users can also choose to incorporate categorical ‘Directory Variables’ which are instead held constant across groups of ROIs (e.g., a group of images from a single site or timepoint). The categories of Directory Variables are derived from folder names and the directory structure in which the images are stored (Figure 2, where Directory Variable 1 and 2 could be site and year, respectively, with consistently named folders).

To permit SizeExtractR to work properly, it is fundamental to set up a consistent system for labeling ROIs, naming folders, and structuring the folder directory (Figure 1). This can be achieved by following three simple steps in preparation for a study using SizeExtractR. (1) If you wish to include Directory Variables, then organize the images in a nested folder directory, and name folders consistently (e.g., Figure 1). (2) Decide on the alphabetical characters you will use to label the different ROI Types for your study (e.g., taxon abbreviations, Figure 2). (3) If you wish to include additional categorical variables, then decide on the alphabetical ROI Label Codes to be used during annotation. This preparation should only take a few minutes.

Finally, the workflow in ImageJ automatically saves three output files per image: a data file with uncalibrated size metrics and ROI name labels, a ROI zip folder to allow later reanalyses, and a reference image showing the annotations to view ROIs quickly and easily (Figure 1).

### 2.2 R‐package

The SizeExtractR‐package contains a series of interactive tools that are used to (1) conduct quality control of image annotations and ROI labeling; (2) add categorical variables (Figure 2) to the size dataset by reading and converting folder names and ROI name labels; (3) calibrate and calculate size metrics; and (4) create a single size dataset for the entire image set, to be saved for further analyses. The functions required to build the size database must be run in a specific sequence. Therefore, to avoid any coding mistakes by the user, an additional R function, Full_SizeExtractR_Workflow(), is included that runs through this entire sequence automatically and requests interactive user input where necessary. The plotting function Plot_Size_Frequency() aids data exploration and presentation. This plotting function can be used to compare size frequency distributions arising directly from the data among different categorical grouping variables (up to three categorical variables implemented). Further size analysis of ROI files can be achieved using the new R‐package: *RImageJROI* (Sterratt & Vihtakari, 2021).

## 3 CASE STUDIES AND WORKED EXAMPLE

SizeExtractR has been developed and used in several research and teaching projects across marine and terrestrial ecology since 2018. These include published studies such as the examination of coral population size structure, heat stress and mass coral bleaching (Lachs et al., 2021), and the determination of size spectra and inferred growth of nursery‐reared and field‐planted corals (Humanes et al., 2021). Several ongoing projects are utilizing SizeExtractR to assess reproductive effort by measuring egg size from microscopy images, quantifying population size structure of coral and sea urchins across large‐scale latitudinal gradients and estimating growth in *Drosophila* flies to assess evolutionary potential under temperature stress. Here we explain the value of SizeExtractR and describe a worked example of the method for the Lachs et al. (2021) case study. A full step‐by‐step example is provided with R‐code in the User Guide (covering both ImageJ and R‐package usage) and as a vignette to the R‐package (covering only the R‐package usage) (Supplementary Materials).

**FIGURE 3:**
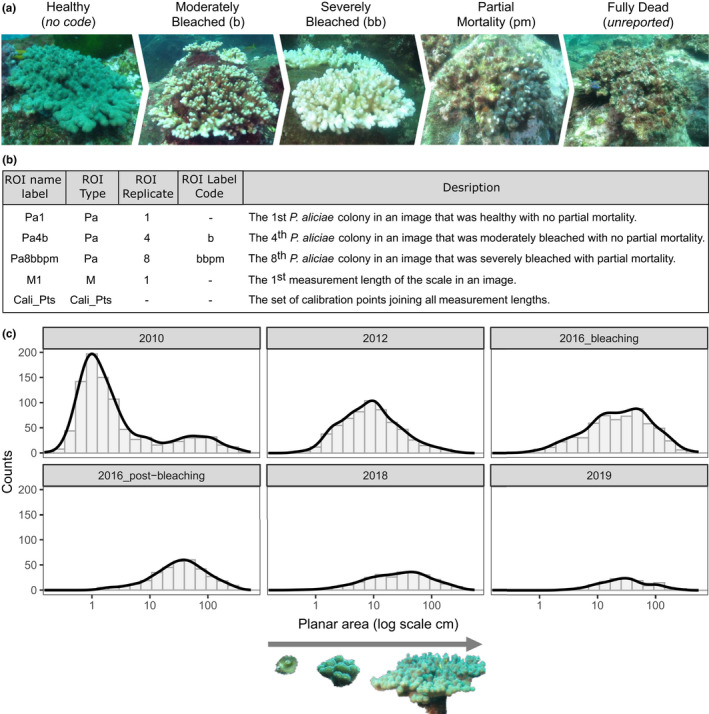
Using SizeExtractR to assess population size structure for hard coral from scaled seafloor photographs of the benthos (Lachs et al., 2021; Sommer et al., 2014). (a) The process of coral bleaching from a healthy state until mortality is shown for *Pocillopora aliciae*. The user‐defined ROI Label Code for recording moderately and severely bleached colonies was b and bb, respectively, for colonies with partial mortality was pm, no ROI Label Code denoted a healthy colony, and dead colonies were not analyzed. (b) Example ROI name labels from this case study are shown with descriptions, including the automatically produced codes for calibration lengths (M) and calibration points (Cali_Pts). (c) Temporal change in population size frequency distributions for *P*. *aliciae* are shown as the direct output of the SizeExtractR plotting function, Plot_Size_Frequency(). Notably, all surveys occurred in Austral winter, except for the 2016 bleaching survey in Austral summer

Lachs et al. (2021) used SizeExtractR to link population size structure, heat stress, and coral bleaching in a regional endemic coral (*Pocillopora aliciae*) in the Solitary Islands Marine Park, eastern Australia. The image dataset comprised of scaled seafloor images (Figure 2—note the calibration stick with multiple 10cm bands) along replicate transects from 2010 until 2019 that encompassed an intense marine heatwave in 2016 with associated mass coral bleaching and mortality (Figure 3a). In planning the image analysis and ROI labeling system for this study, it was important to consider which variables were to be recorded. The size metric of interest was planar area (cm^2^), easily captured using SizeExtractR‐macros. The categorical Directory Variables of interest were year (6 years), site (4 sites/year), and transect (3 transects/site/year). Thus, the folders were named consistently and placed in a nested structure (images within transect folders, within site folders, within year folders, within a single root directory). Finally, the categorical ROI Variables of interest were bleaching status (healthy, moderately bleached, and severely bleached), and partial mortality, given that partial mortality of colonial organisms can occlude size‐age relationships (Figure 3a). Accordingly, the ROI labelling system used for annotations reflected these user‐defined categorical variables with simple codes (Figure 3b). Note, these labelling codes would be different for every study.

In this example, the SizeExtractR plotting function, Plot_Size_Frequency(), was used to compare size frequency distributions through time quantitatively (Figure 3c). A spike in the abundance of small *P*. *aliciae* colonies occurred in 2010, which increased through the size classes until 2016 representing cohort growth, without additional recruitment in other years (Lachs et al., 2021). The statistical significance of these trends was then tested further in R using generalized linear mixed effects models. Such size datasets can be used for numerous other analyses including timeseries analysis, or growth estimation (multiple size surveys of individuals through time) and population projections (e.g., integral projection modelling).

## 4 TIME SAVING WITH SizeExtractR

To quantify the time saving benefits of using SizeExtractR‐macros, we conducted an image analysis time trial on a subset of benthic images (*N* = 40) from Lachs et al. (2021), compared to a fully manual method using default ImageJ tools. Briefly, the manual method involves opening an image in ImageJ, selecting the outline tool, outlining a coral, adding the outline to the ROI manager, renaming the ROI, and repeating for each coral in the image, switching between ImageJ tools each time by navigating through dropdown menus. Once each ROI is named correctly, the size metrics are chosen from a drop‐down menu, results are exported to a text file, ROIs saved to a zip file, and a reference image is formatted and saved, with all output file names typed in manually. Each image was analyzed using both methods, all corals per image were annotated, and the image analysis time per image was recorded (see data in Supplementary Materials). Data were analyzed using general linear models (see full methodology in the Supplementary Materials).

**FIGURE 4:**
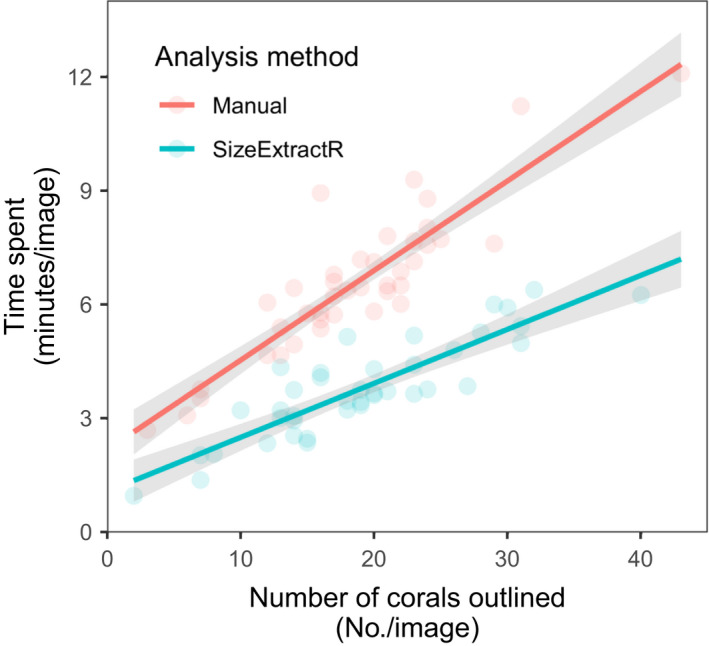
Comparison of time spent on ImageJ analysis between the SizeExtractR method and a manual method, tested using generalized linear model (mean ± 95% confidence interval). The manual method (red) uses default ImageJ tools to produce ROI size measurements, while the SizeExtractR method (blue) uses custom SizeExtractR‐macros to speed up the image analysis workflow. While time spent per image is directly proportional to the number of corals outlined (per image), the SizeExtractR method took approximately half the time than the manual method with default ImageJ tools, for a given number of corals per image

Time spent per image was found to increase linearly with the number of corals per image for both analysis methods (Figure 4). Importantly, the SizeExtractR method was up to 49% faster than the manual method, whereby the amount of time saved increased with the number of objects per image (i.e., significant interaction term, Table A1). The additional time saving for densely packed images (i.e., many objects of interest) when using SizeExtractR was most likely due to additional observer fatigue for manual methods. Specifically, the manual method requires users to search through drop‐down menus to set tools in ImageJ and navigate pop‐up boxes to save output files; steps that are automated in SizeExtractR‐macros. Together, our results show the SizeExtractR method took approximately half the time of the manual method with default ImageJ tools, for a given number of corals per image. Moreover, human errors in annotating ROIs and saving output files (e.g., spelling mistakes or overwriting data files) were common using the manual method (~15 min of careful quality control was needed after ~2 h of annotation), but near non‐existent when using Size Extract R.

## 5 SUMMARY

In ecology, there is a growing need for tools and workflows that allow for reproducible extraction of object sizes from scaled images (Weinstein, 2018). Despite advancements in flexibility and accessibility in recent years, machine learning image analysis techniques are often unsuitable for specific image datasets, either due to difficulties in adapting models to new purposes, budget, timeframe, or level of expertise required. Here, we present SizeExtractR as an adaptable workflow solution to these problems. SizeExtractR comprises a user‐friendly ImageJ‐macro and an R‐package for measuring the dimensions of irregular shaped objects in scaled images using seven common size metrics. Combining the accuracy of human recognition with the speed of semi‐automated protocols, SizeExtractR sits between fully manual image analysis methods (e.g., ImageJ default tools, Schneider et al., 2012) and automated machine learning image analysis methods (e.g., CoralSeg, Alonso et al., 2019). SizeExtractR offers high levels of transparency and traceability (in terms of fully documented outputs including ROI outline images) and quality control to researchers. By providing automated saving of output files, SizeExtractR can facilitate the scientific peer review process and allows researchers to re‐check or add to their earlier image analysis work. Future directions include the inclusion of additional size metrics and improving the user interface for the R‐based portion of the workflow (e.g., an interactive Shiny App). SizeExtractR is open source, offers features that promote openness, and replicability in science, and has numerous potential applications across ecology, evolution, botany, and other scientific disciplines.

## CONFLICT OF INTEREST

The authors declare that we have no conflict of interest.

## AUTHOR CONTRIBUTIONS

**Liam Lachs:** Conceptualization (lead); Data curation (lead); Formal analysis (equal); Funding acquisition (equal); Methodology (lead); Project administration (lead); Resources (lead); Software (lead); Visualization (lead); Writing – original draft (lead); Writing – review & editing (lead). **Fiona Chong:** Data curation (supporting); Formal analysis (equal); Methodology (supporting); Software (supporting); Writing – review & editing (supporting). **Maria Beger:** Conceptualization (supporting); Funding acquisition (equal); Methodology (supporting); Project administration (supporting); Supervision (equal); Writing – review & editing (supporting). **Holly K. East:** Supervision (supporting); Writing – review & editing (supporting). **James R. Guest:** Project administration (supporting); Supervision (equal); Writing – review & editing (supporting). **Brigitte Sommer:** Conceptualization (supporting); Funding acquisition (equal); Methodology (supporting); Supervision (supporting); Writing – review & editing (supporting).

### OPEN RESEARCH BADGES

This article has earned an Open Materials Badge for making publicly available the components of the research methodology needed to reproduce the reported procedure and analysis. All materials are available at https://doi.org/10.25405/data.ncl.15106455.

## Supporting information

Supplementary Material

## Data Availability

All data and scripts developed for this study are publicly and freely available. The SizeExtractR workflow R‐package is available as a Github release (https://doi.org/10.5281/zenodo.5997934) and the ImageJ‐macros, User Guide, and underlying data and R code for the timed image analyses are available as a data repository (https://doi.org/10.25405/data.ncl.15106455). Both are also linked from the CORALASSIST Lab website (https://www.coralassistlab.org) and the first author's github page (https://github.com/liamlachs/SizeExtractR).

## Time Trial Methods and Statistical Results

We conducted an image analysis time trial on a subset of benthic images (*N* = 40) from Lachs et al. (2021) to quantify the timesaving benefits of using SizeExtractR‐macros, compared to a fully manual method using default ImageJ tools. Each image was analyzed using both methods, all corals per image were annotated, and the image analysis time per image was recorded. Half of the images were analyzed with SizeExtractR first, while the other half were analyzed using the manual method first, to check for issues relating to recognition of an image previously analyzed. After collating the data using the SizeExtractR, the total number of coral colonies was computed per image. Notably, the image analysis workload was shared between two observers (L.L. and F.C.). The degree to which time spent on analysis (per photo) (continuous response) was affected by the analysis method (categorical predictor), the number of corals per photo (numerical predictor) was tested using a general linear model. Observer ID (categorical predictor) and Method First (categorical predictor) were additional potential sources of variation that were included in the full model. The predictors in this model were then reduced using backward selection of non‐significant predictors one at a time.

**TABLE A1:** Results of multiple regression relating to Figure 4 in the main manuscript, showing the relationship between time spent per image (continuous response) and various covariates: analysis method (categorical predictor), number of corals per image (continuous predictor), their interaction, and other sources of potential error observer ID (categorical predictor), and Method First (categorical predictor). The reduced and then final models were formed after backward selection removal of non‐significant predictors starting from the full model

Model	Predictor	Estimate	*SE*	*T* value	*p* Value
Full	Intercept	2.29	0.39	5.86	**<.001**
	Method‐SizeExtractR	0.23	0.02	13.15	**<.001**
	No. corals	−1.13	0.45	−2.51	**<.05**
	Observer.ID‐ L.L.	−0.24	0.19	−1.28	>.05
	Method. First‐SizeExtractR	0.29	0.17	1.76	>.05
	No. corals*Method‐SizeExtractR	−0.09	0.02	−4.12	**<.001**
Reduced	Intercept	2.88	0.31	9.17	**<.001**
	Method‐SizeExtractR	0.20	0.01	14.53	**<.001**
	No. corals	−1.48	0.38	−3.93	**<.001**
	Observer.ID‐Lachs	−0.26	0.16	−1.65	>.05
	No. corals*Method‐SizeExtractR	−0.07	0.02	−3.64	**<.001**
Final	Intercept	2.61	0.27	9.75	**<.001**
	Method‐SizeExtractR	0.21	0.01	15.96	**<.001**
	No. corals	−1.48	0.38	−3.91	**<.001**
	No. corals*Method‐SizeExtractR	−0.07	0.02	−3.62	**<.001**

The *p* Values of significant predictors for on alpha level of .05 are shown in bold.
