# Supplementary material for: SizeExtractR: A workflow for rapid reproducible extraction of object size metrics from scaled images
Source: Ecol Evol. 2022 Mar 14;12(3):e8724. doi: 10.1002/ece3.8724 (PMC8928875; doi:10.1002/ece3.8724)
Supplement: Supplementary file 1 — Supplementary Material [file ECE3-12-e8724-s001.pdf]

SizeExtractR User Guide
(ImageJ-macro & R-package)

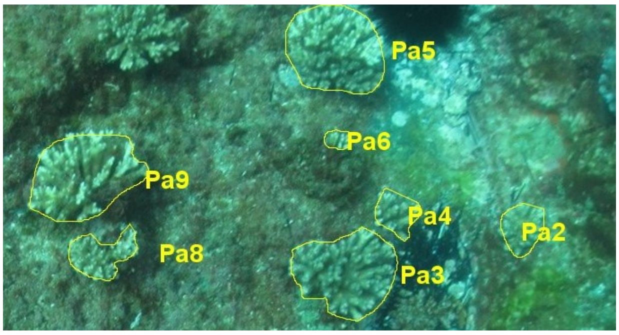

Contents

Introduction 2

Part 1 – One-off Setup of SizeExtractR 4

Part 1.A - ImageJ Download 4

Part 1.B - Macro Installation 4

Part 1.C – R Download 5

Part 1.D – SizeExtractR R-package Installation 5

Part 2 - ImageJ Analysis in ImageJ 6

Part 2.A – Decide on a Labelling System 6

Part 2.B – Directory Variables & Organising Images 7

Part 2.C --- ROI Variables (ROI Type, ROI Replicate, & ROI Label Code) 8

Part 2.D – Step-by-step Workflow - Macros and Shortcuts 10

Part 2.E – Additional Other Tools (macros and shortcuts and links) 12

Part 3 – Calibrated Database Formation in R 13

Part 3.A – Checking, Collating, and Building a Calibrated Dataset 13

Part 3.B Worked Example using the main SizeExtractR workflow function 13

Part 3.B.I - Prerequisites 14

Part 3.B.II - Organising raw images and directory tree 14

Part 3.B.III - Choosing a user-defined labelling system 14

Part 3.B.IV - Image analysis using ImageJ macros to export size uncalibrated size data 15

Part 3.B.V - Quality control, compiling text files to single size dataset, and saving in R 16

Part 3.B.VI - Plotting size-frequency distributions in R. 21

Part 3.C – Step-by-step without using the all-in-one- wrapper function 22

Part 3.D – Additional Functions (Full Workflow Function and Plotting Function) 24

SizeExtractR User Guide

Introduction

This methodology is designed to be used when:

- Multiple objects must be measured within each image. These objects are called Regions of Interest (ROIs).
- There are a huge number of images, so fully manual image annotation will be slow. SizeExtractR automates some labour-intensive parts of this workflow.
- Each image is scaled (i.e., includes a calibration of known length)

Useful Size Metrics extracted from images using this protocol are:

- Area (of irregular or regular-shaped ROIs)
    - Circular equivalent diameter (derived from the area measurement)
    - Extruded spherical volume (Derived from circular equivalent diameter)
- Maximum/Minimum Feret's Diameter (equivalent to diameters measured with calipers)
    - Geometric mean diameter (derived from mean of Max/Min Feret)
- Perimeter length.
- Additional user-defined categorical variables that relate to individual ROIs (e.g., health status)
- Size Frequency Distributions by grouping variable (up to two variables allowed)

Useful Additional Metrics that can be easily derived are:

- Total imaged area
- ROI density per unit area
- Population size per area imaged (total number of ROIs)

Background:

This workflow is described using an example of a coral dataset (see Lachs et al. 2021). Scaled images of the seabed were taken during annual field surveys at 4 sites, with 3 transects per site, that have been surveyed since 2010 (Transect within Site within Year). Each image contained up to 40+ individual coral colonies. We were interested in the size of individual coral colonies after a mass bleaching event occurred in 2016 (see example in Fig. 1).

W**orkflow overview**:

1. Prepare a set of scaled images into a consistent nested directory structure (e.g., images within transect folders within site folders within year folders within one overall directory).
2. Decide on a labelling scheme for the ROI name label codes (e.g., c = coral, b = bleached).
3. Using the SizeExtractR ImageJ-macro, analyse all images by outlining the objects of interest (corals), naming them according to the labelling scheme, and measuring calibration lengths.
4. Using the SizeExtractR R-package, first perform some checks to identify any potential human errors that occurred during labelling, amend those errors, then collate all data files into one calibrated database.

SizeExtractR provides simple functions in both ImageJ and R to perform this workflow.

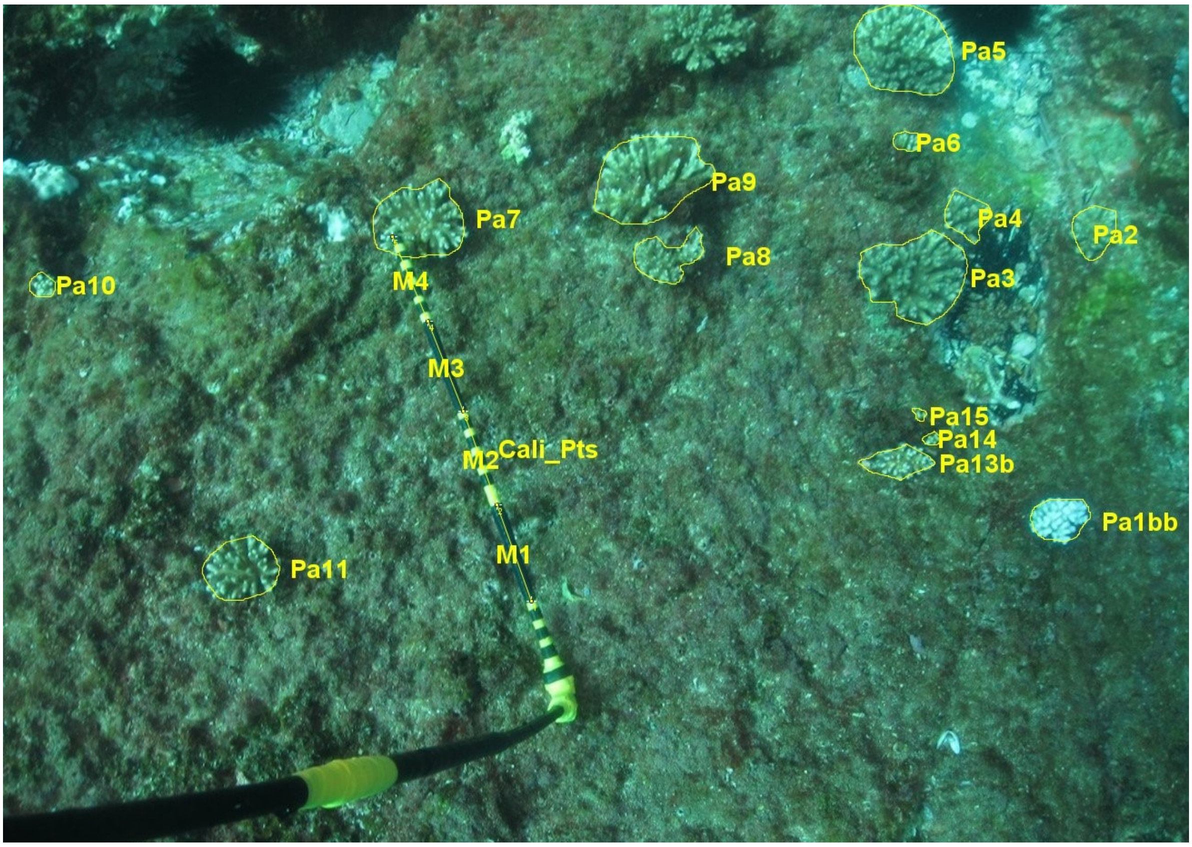

Figure 1. Scaled image from the example dataset showing corals of different sizes at different levels of bleaching from 2016, North Solitary Island, eastern Australia (Lachs et al. 2021). The calibration stick is broken down into 10cm lengths.

SizeExtractR User Guide

Part 1 – One-off Setup of SizeExtractR

The following subsections are:

A. ImageJ download
B. SizeExtractR ImageJ-macro installation
C. R Download
D. SizeExtractR R-package installation

Part 1.A - ImageJ Download

Download ImageJ either as the basic programme (https://imagej.nih.gov/ij/download.html), as the Fiji distribution (https://imagej.net/Fiji/Downloads). Funnily enough, Fiji doesn't install like a regular programme. It is an App. So you just:

1) download the zip folder.
2) Extract all contents of this folder to a folder in your "My Documents" (or wherever).
3) You will find the Application file "ImageJ-win64" (e.g for Windows) within the main folder for Fiji.
4) Double click on this to launch ImageJ – no installation needed.

SizeExtractR will also work on the basic ImageJ programme (not Fiji).

Part 1.B - Macro Installation

Background:
Why use Macros? and What are they?

Macros are programmed ImageJ workflows that serve to speed up the image analysis by automating processes. Macros can be initiated by tapping specific keyboard shortcuts (one for each SizeExtractR macro).

Working in ImageJ can be quite slow. Particularly, switching between tools and saving files can become an extremely laborious task.
For one image you need to choose: hand tool (to pan in the image), freehand polygon tool (to outline the object of interest and make an ROI), add and rename the ROI (with the user-defined labelling scheme), line tool (to measure calibration lengths), and REPEAT many times.
At the end of each image you must then save all relevant measurements in a sensible way.

We don't want to have to think too hard whilst outlining/measuring ROIs. We want to be able to sit back, put on some good music, with nice shortcuts that optimise the process as much as possible. Once you find a good rhythm it is easy!

To make the entire process more fluid we have written various ImageJ macros. These tools speed up the image analysis by about a factor of two (see main manuscript), reduce human error considerably, and simplify the workflow and make it more efficient and less error prone. These macros are saved in the Startup Macros script in ImageJ. If you are interested to see this code then you can work on it there. For the advanced user, descriptions of all macro functions can be found in the directory @ https://imagej.nih.gov/ij/developer/macro/functions.html

NOTE: Avoid changing the macro scripts. They are very sensitive to any change and may stop working if edited. Feel free to contact me if you want to make any changes @ liamlachs@gmail.com.

SizeExtractR User Guide

Installation Guide:

Open the Startup Macros script:

`Plugins > Macros > Startup Macros`

The macros contained in this ImageJ script load every time ImageJ is run. If we want to embed our new shortcuts within ImageJ (which speed things up very nicely), they must be appended to this startup macro script.

1) Download the SizeExtractR ImageJ macro script, found at the data repository https://doi.org/10.25405/data.ncl.15106455 as the only text file in the ImageJ_macro_code.zip archive.

2) Open the `SizeExtractR_ImageJ_Macro.txt` file which holds the script for new shortcuts. Copy all text.

3) Paste this text into the very end of the ImageJ Startup Macros script starting on a new line.

4) Now save this new script (Ctrl+s).

5) Close and re-open ImageJ for the SizeExtractR macros to take effect.

6) You can now see the SizeExtractR shortcuts listed under `Plugins > Macros`

7) For a full description of how to use each of these very useful tools please see next section

Part 1.C – R Download

SizeExtractR R-package was built on and is recommended to be used with R version 4.

This can be downloaded from:

https://www.r-project.org/

Part 1.D – SizeExtractR R-package Installation

The R-package `devtools` is needed to install SizeExtractR, and must be installed first. Then to install SizeExtractR, run the following:

```
library(devtools)

devtools::install_github("liamlachs/SizeExtractR")
```

Accept relevant package dependency installations and updates.

To use SizeExtractR load it using:

```
library(SizeExtractR)
```

SizeExtractR User Guide

Part 2 - ImageJ Analysis in ImageJ

A large monitor and a mouse are recommended to make outlining objects easier. Note that the first three section give background on SizeExtractR, and the fourth section gives a step-by-step user guide to running the image analysis protocol.

The following subsections are:

A) Decide on a Labelling System
B) Directory Variables & Organising Images
C) ROI Variables (ROI Type, ROI Replicate, & ROI Label Code)
D) Step-by-step Workflow - Macros and Keyboard Shortcuts
E) Additional Useful Tools (macros and shortcuts)

Part 2.A – Decide on a Labelling System

In the final calibrated dataset output in R, each row represents a different ROI (i.e., object of interest or calibration length). The different variables (i.e., columns in the dataset) describe these ROIs. The numerical variables that are measures of size (e.g., area, max/min diameter) are produced automatically in ImageJ. However, some additional categorical variables that are user-specific can also be incorporated.

There are two types of categorical variables.

1) **Directory Variables:**
   Variables that are <u>the same for all ROIs within a single image</u> (e.g., transect, site, or year). SizeExtractR R-package derives these variables from the folder names in the directory containing the images, therefore ensuring all folders are named correctly is of utmost importance. We refer to these as Directory Variables.

2) **ROI Variables**:
   Variables that can be <u>different for each ROI within a single image</u> (e.g., ROI Type: calibration length or object of interest, like a coral, or ROI Code: user-defined variable to give extra information about that ROI, like whether the coral is bleached or not). The SizeExtractR R-package derives these from the ROI name label assigned during image analysis in ImageJ. We refer to these as ROI variables, and there are two types of these.
   a. ROI Type – this is either the Calibration Point (“Cali_Pts”), the Calibration Length (“M”), or the object of interest (a user-defined alphabetical code – e.g., “c” for coral). This distinguishes the calibration ROIs from the ROIs of interest (i.e., corals), and <u>makes up the beginning of the ROI name label</u>.

   b. ROI Replicate – this is a unique number per ROI that identifies each ROI of a specific ROI Type within a single image. For instance, the name label for the 1st coral outlined in the image would be “c1”. The second coral outlined in that image would be “c2” and so on. See below for more details.

   c. ROI Label Code– this is any set of user-defined variables (e.g., “b” for bleached, or nothing for not bleached). This is used to record extra information about the ROIs during image analysis and <u>makes up the end of the ROI name label</u>.

Part 2.B – Directory Variables & Organising Images

For full integration with the R script, images must be organised in a logical consistent directory structure. The folders holding different images must be named consistently, as the R-package derives Directory Variables from the folder names of the directory.

For instance, annual photo-quadrat surveys of coral reefs at three sites from 2010-2012 would need to have a directory structure and consistent naming system like so:

Table 1. showing folder names in a nested computer directory.

Directory Level 0		Directory Level 1		Directory Level 2
Photo_Surveys	→	2010	→	Site_1
			→	Site_2
			→	Site_3
	→	2011	→	Site_1
			→	Site_2
			→	Site_3
	→	2012	→	Site_1
			→	Site_2
			→	Site_3

Setting this directory in R would then use the following string which would then access all images from all the ‘Site’ subfolders across all years:

```
dir <- “C:/users/<user name>/Photos_Survey”
```

Do not use the “~” symbol as a replacement for the full current directory string, as this will cause an error. Note that this User Guide uses examples from Windows but that it will also work on other platforms (e.g., Mac).

If Directory Variables are not needed for a specific study, then all images can be pooled in a single folder.

Part 2.C --- ROI Variables (ROI Type, ROI Replicate, & ROI Label Code)

A consistent ROI labelling scheme is essential, as the R-package will use these to form the calibrated size database. The labelling scheme is different for the three categories of ROIs that we use. These ROI categories are:

1. Objects of Interest (e.g., a coral)
2. Measurement Lengths (for Calibration)
3. Calibrations Points (a shortcut for consecutive calibration lengths – see Part 2.A – ROI Variables)

1) Objects of Interest: The ROI name label is a concatenation of three sections. These sections are inputted in order directly after one another without any separator. These three sections are:

i. *ROI Type:* This is a categorical ROI Type Variable which must have at least one grouping level. Note that multiple grouping levels are allowed too. For example, if we are only interested in corals the only code needed is "c" for coral. However, if we are interested in multiple taxa, then this could be recorded as:
   - c - coral
   - s - sponge
   - u - sea urchin

   NOTE: The code "M" cannot be used as this is reserved for calibration measurement lengths.
   NOTE: Capital and lower case letters are treaded differently. You can use only one letter if you wish. Numbers and special characters are not allowed, as numbers are for the ROI replicate.

ii. *Replicate Number in that Group:* The number of that ROI of that group in that specific image. i.e. the 4th coral outlined in that photo would be recorded as = c4
   Note that it is okay if ROIs from different images have the same code (e.g., multiple images that contain the ROI code c4).

iii. *ROI Code:* This is a string of the additional user-defined variables to define. For instance, if we want to record bleaching and partial mortality for each coral then we would do the following:

   Bleaching
   - Not Bleached (Healthy) - Code = "*leave blank*" - i.e., c4
   - Bleached (white) - Code = b - i.e., c4b

   Partial Mortality
   - No partial mortality (Healthy) - Code = "*leave blank*" - i.e., c4
   - Partially dead - Code = pm - i.e., c4pm

   EXAMPLES: The 3rd coral in the photo. It is bleached and has partial mortality: c3bpm
   The 4th coral in the photo. It is not bleached and has partial mortality: c4pm etc.
   NOTE: no label can be used to code for multiple different categorical variables.

2) Calibration Length: Made either using either using macros [7] + [8] OR macros [p] + [u].
Code Names: either M1, M2, M3 or M4 (M for measured length). M0 or M with a value greater than 4 (e.g., M5) is not allowed.

3) Calibration Points: This is an ROI that is created when making the Measurement Length (M1, M2, M3, M4) using macros [7] + [8]. This ROI has no other use than creation of M1-M4 which are used later in R to calibrate the size metrics.
Code Name: Cali_Pts

Part 2.D – Step-by-step Workflow - Macros and Shortcuts

As mentioned before, SizeExtractR macros are found in `Plugins > Macros > Startup Macros`.
NOTE 1: the order of the macro descriptions is different in this manual than in the startup macros script – so they can be explained in a more logical way.
NOTE 2: the hotkey for the keyboard shortcut is found in the square brackets [ ] (i.e. hand tool = press the "g" key)

For the following step-by-step, you can try it yourself on some sample images. Download an unpack the data repository (https://doi.org/10.25405/data.ncl.15106455). Open the folder "Data_preImageJ" and look inside. This folder contains some example images for you to annotate. The final version of fully annotated images is found in the folder "Data_postImageJ_all". That is how your analysis should look once the ImageJ step is complete.

1) Open ImageJ, then:
   > `Ctrl + o` (Command key on Mac, opens popup box to open the first image to be analysed)
   > (Find the appropriate folder that contains the images for analysis)
   > (to start analysis in a new folder [or continue where you left in an unfinished folder], double click on the first [appropriate] image file in the list).

2) For each image in that folder, follow the following protocol. Then move to the next folder of images:

   i. **Name** - "Setup For Benthic Plot Analysis [l]"
   Shortcut Key - `l`
   Use - This macro sets the size metrics to be measured and records, and opens the ROI manager panel which is useful to have open whilst working.

   ii. **Name** - "multipoint line [7]"
   Shortcut Key – 7
   Use - For calibration.
   Pick the points to be used to make calibration lengths (see shortcut [8]).

   iii. **Name** - "Calibration Lengths [8]"
   Shortcut Key – 8
   Use - For calibration.
   Saves the output of multipoint line [7] as separate lengths called M1, M2, M3, M4. It saves the lengths between consecutive points. Hence the placement of points using multipoint line [7] must be linear and consecutive.
   NOTE: You will specify the true calibration length (in users own units, e.g., centimetres) later in R software.
   NOTE: You cannot move onto the next image until a minimum of 1 calibration length has been save as an ROI (named M1, M2, M3 or M4).

   iv. **Name** - "freehand [q]"
   Shortcut Key – q
   Use - This tool draws a freehand polygon. Used to outline the ROIs of interest (e.g., corals).

v. **Name** - "AddAndNameROI [u]"
Shortcut Key – u
Use - Used to add the ROI to the ROI manager, and give it a ROI name label, in accordance with the user-defined labelling scheme.
A box will appear asking for a name. Here you must input a string that the R-package has been programmed to read. For a full description of the string see below.
NOTE: This function is designed to disallow ROIs with the same name. Therefore, if we are working on corals, and in the image there are three coral colonies, then they must be named: c1, c2, c3. This helps to identify individual colonies at a later stage.

vi. **Name** - "Save Area Next Image [n]"
Shortcut Key – n
Use - This function is designed to be run once you are finished both outlining ROIs AND measuring calibrations lengths.
This function saves:

a. all ROIs in a zip folder that can be reopened (along with the image) if you want to add new ROIs to the image after.
b. a text file containing all data including size metrics and ROI name labels.
c. an outlines JPEG image showing all ROIs - so users can easily review ROIs at a later stage without having to reopen ImageJ.

NOTE: The output text file, ROI zip folder and JPEG file are saved in an auto-created folder called “ImageJ_Output”. That folder must not be edited as it will be read by the R-package.
NOTE: To ensure you do not accidentally overwrite the data (.txt, .zip, outlines.jpg). If you want to overwrite data you must check the yes in the popup box (it will ask twice).
Finally the function clears the ROI manager panel and Results panel and moves to the next image.
NOTE: The function will only run if a minimum of 1 calibration lengths ROI is present (Calibration lengths called either M1, M2, M3 or M4).

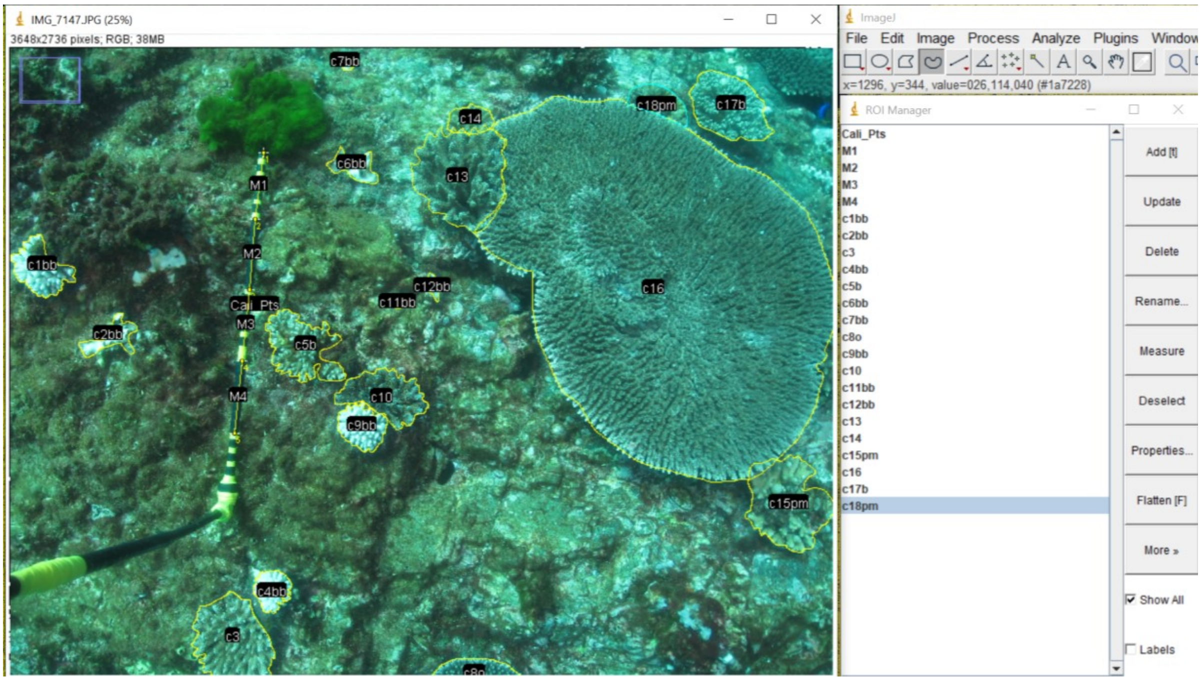

Figure 2. Image analysis screenshot with all labelled ROIs added to the ROI manager, showing calibration points (*Cali_Pts*), 10cm calibration lengths (*M1* to *M4*), and coral ROIs named according to the labelling scheme (*c*: coral, *b*: moderately bleached, *bb*: fully bleached, *pm*: partial mortality, and *o*: out-of-frame). Once the image is completed to this level, the user needs to use the shortcut 'Save Area Next Image [n]' to save all relevant files and move to the next image in the folder.

Part 2.E – Additional Other Tools (macros and shortcuts and links)

1) **Name** - Ctrl and scroll (zoom on mouse) (Mac: + and – sign to zoom in and out)
   Use - Zoom in/out. Use a magnification that allows you determine the objects of interest.

2) **Name** - "hand [g]"
   Shortcut Key - g
   Use - Set the hand tool to pan across image when zoomed in.

3) **Name** - "line [p]"
   Shortcut Key – p
   Use - For calibration.
   Line tool to draw a line between two points. This must be used in conjunction with AddAndNameROI [u] to name the ROI as a calibration length (M1, M2, M3, or M4).
   Note that for most cases the shortcuts [7] and [8] will be more useful.

4) **Name** - "Next image - no change [r]"
   Shortcut Key – r
   Use - This is useful for a folder full of original jpgs, outline jpgs, zip folders and txt files. The shortcut [n] (above) will move on to the next image which will be an outlined image without ROIs. When this happens, use this shortcut [r].
   It will automatically open the next image (the next original image to be analysed) and all ROIs that go along with it. You can then add ROIs as you wish.

5) Further size analysis of ROI files can be achieved using the new R-package: *RImageJROI* (Sterratt and Vihtakari, 2021).

Part 3 – Calibrated Database Formation in R

Three main steps in R are described here in subsections. The following subsections are:

A. Checking, Collating, and Building a Calibrated Dataset
B. Worked Example using the main SizeExtractR workflow function
C. Step-by-step without using the all-in-one- wrapper function
D. Additional Functions (Full Workflow Function and Plotting Function)

Part 3.A – Checking, Collating, and Building a Calibrated Dataset

Within this R package there are series of seven R functions that are used to check for human errors made during image analysis, for instance an ROI name label being written incorrectly, to build an error free calibrated dataset. These are designed to all be run in sequence.

There is an additional function which runs these seven in-sequence functions automatically, however, for the purposes of learning, <u>it is worth first running the function individually</u>.

For all R functions, you can use the help file to understand the specific inputs needed, and outputs.

```
? <name of function>
```

Note that during data checks you may be shown a mistake (e.g., made during ROI labelling, or folder naming). This will cause R to error and provide you with information on the terminal about what the problem was during quality control checking, and how to fix it. The user may need to fix mistakes outside of the R environment. Then, simply re-run that R function to check if it now passes the quality control check.

<u>**Likely Errors and how they can be fixed:**</u>

1. <u>**Directory Variables**</u>
   If you find a mistake in the directory variables, this either relates to a misspelling in a folder name, or an inconsistent directory structure (e.g., if Site folders are always within Year folders, but in one case it is not, this will flag an error). You will need to fix this.
   To do that, rename / organise the folders appropriately outside of the R environment.

2. <u>**ROI Type and ROI Code Variables**</u>
   If you find a mistake during ROI labelling, then you will need to fix this.
   To do that, within ImageJ open the offending image and the zip file containing the ROIs for that image. Open the image, then clear the ROI manager, and then open the ROI zip file. Once that is complete, rename the ROI from the ROI manager panel, then save the files using shortcut [n].

Once all these quality control checks are passed, you should have a calibrated size dataset. Congratulations!

Part 3.B Worked Example using the main SizeExtractR workflow function

To help users to learn the full protocol, this section of the user guide describes a fully worked example based on a subset of data from Lachs et al. (2021), provided at the data repository (https://doi.org/10.25405/data.ncl.15106455). This database contains images of the seabed, each including a measurement scale. The images were taken during and 6-months after a coral bleaching event in 2016 in the Solitary Islands Marine Park, Southeast Australia. The aim here is to assess how population size structure of the coral *Pocillopora aliciae* differs before and after coral bleaching event.

SizeExtractR User Guide

This worked example follows through all the main sections of the SizeExtractR workflow as shown by the subsections:

I. Prerequisites
II. Organising raw images and directory tree
III. Choosing a user-defined labelling system
IV. Image analysis using ImageJ macros to export size uncalibrated size data
V. Quality control and compiling all datafiles into a single size dataset and saving in R
VI. Plotting size-frequency distributions in R

Part 3.B.I - Prerequisites

First software must be installed and prepared. ImageJ must be installed locally (see Part 1.A) and the SizeExtractR macros attached as start-up macros (see Part 1.B). R must be installed locally (see Part 1.C) and the SizeExtractR R-package must be installed (see Part 1.D). There are two datasets for the worked example (https://doi.org/10.25405/data.ncl.15106455):

- Before ImageJ: The first is simple a folder of raw images, named "Data_preImageJ". This will be used to learn how to use the ImageJ macros on a set of raw images.
- After ImageJ: The second is the same folder of images, except after the ImageJ analysis, named "Data_postImageJ_all".

Explore these folders now to get an idea of how the output files from ImageJ look.

Part 3.B.II - Organising raw images and directory tree

First the images must be organised within a single folder or in a directory tree manually. This has already been done in this worked example. Open the folder "Data_preImageJ" and explore this directory. Note that we have two levels in this directory:

1. Timepoint folders entitled:
   - 2016_during-bleaching
   - 2016_post-bleaching
2. Site folders (referring to two of the islands in the Solitary Islands Marine Park) entitled:
   - North
   - Northwest

Note that the naming of these folders must be consistent throughout the directory levels. For this case, the first directory level has only "year" folders, and second directory level has only "site" folders, named consistently within each year folder. The images are then all stored in the "site" folders.

Part 3.B.III - Choosing a user-defined labelling system

As described in the manuscript, the ROI name labels that are annotated for each object in each image are a combination three codes ROI Type, ROI Replicate, and ROI Label Code. For this example we will are only interested in the coral *P. aliciae*, so the ROI Type code is 'Pa'. Now we must define a simple labelling system for the ROI Label Code (the final alphabetical code in the annotation). In this example, we will record partial mortality and bleaching status (moderate or severe) as in the example shown in the SizeExtractR manuscript (Fig. 4). The labelling system can be seen in Table 4.

Table 4. User-defined ROI Label Code labelling system. Note that a coral can also be labelled without any of these factors, for example a healthy coral could be labelled as Pa1, where a moderately bleached coral would be Pa1bb. This table is also what will be used later during quality control in R.

ROI_Label_code	Corresponding_Variable_Name
b	Mod_Bleached
bb	Sev_Bleached
pm	Partial_Mortality

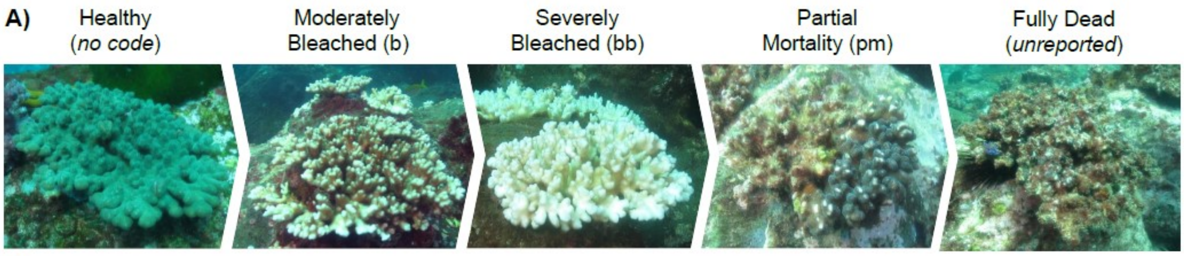

B)

ROI name label	ROI Type	ROI Replicate	ROI Label Code	Desription
Pa1	Pa	1	-	The 1st *P. aliciae* colony in an image that was healthy with no partial mortality.
Pa4b	Pa	4	b	The 4th *P. aliciae* colony in an image that was moderately bleached with no partial mortality.
Pa8bbpm	Pa	8	bbpm	The 8th *P. aliciae* colony in an image that was severely bleached with partial mortality.
M1	M	1	-	The 1st measurement length of the scale in an image.
Cali_Pts	Cali_Pts	-	-	The set of calibration points joining all measurement lengths.

Figure 4. Using SizeExtractR to assess population size structure for hard coral from scaled seafloor photographs of the benthos (Lachs et al., 2021; Sommer et al., 2014). A) The process of coral bleaching from a healthy state until mortality is shown for *Pocillopora aliciae*. The user-defined ROI Label Code for recording the different categories are shown in backets. Moderately and severely bleached colonies are recorded as ‘b’ and ‘bb’, respectively, and partial mortality is recorded as ‘pm’. No ROI Label Code denoted a healthy colony, and dead colonies were not analysed. B) Example ROI name labels are shown with descriptions, including the automatically produced codes for calibration lengths (M) and calibration points (Cali_Pts). Adapted from Manuscript Figure 3.

Part 3.B.IV - Image analysis using ImageJ macros to export size uncalibrated size data

Now, we will demonstrate the image analysis on one image from the dataset of raw images (folder name is “Data _pre-ImageJ”). However, this would then be repeated for each image in the dataset.

Follow the steps below:

1. Open ImageJ.
2. Press the shortcut [l] to make the ROI manager panel appear.
3. Open the image you want to analyse.
4. To measure the 10cm lengths on the measurement stick:
    - Press the shortcut [7] to select the multipoint line tool.
    - Using the mouse click on each border between the 10cm lengths of the measurement stick.
    - Once five points are shown press the shortcut [8] to save the measurement length
    - Notice the new ROIs stored in the ROI manager.

5. To outline and annotate the first coral:
   - Press the shortcut [q] to make sure the freehand tool is selected.
   - Manually outline a coral using a mouse click and drag.
   - Press the shortcut [u] and type in the ROI name label for that coral (e.g., if it is the first coral in the image to be annotated and it is a severely bleached coral, this will be 'Pa1bb').
6. Repeat step 5 for every other coral in the image.
7. To save extract the size data, save all output files, and move to the next image in this folder, simply press the shortcut [n].

Now you have finished the analysis on the first image. On your own dataset this would be completed for all the other images in the dataset, however for the sake of this example you can stop at this stage and continue to the next step. The second dataset folder, named "Data _post-ImageJ_all", is a fully completed analysis for this image dataset, with annotations for all corals and all images.

Part 3.B.V - Quality control, compiling text files to single size dataset, and saving in R

Now we will work from the processed image dataset, named "Data _post-ImageJ_all". The next step is to complete the quality control of annotations and build the single size dataset based on the data from each image which are currently stored as individual text files in the subfolders named "ImageJ_Output". The code can be copied into and run from an R script to run through the example.

Follow the steps below:

- **Setup**
  1. Open R.
  2. Install SizeExtractR: Do this only if the package is not yet installed.
     ```
     > library(devtools)
     > devtools::install_github("liamlachs/SizeExtractR")
     ```
  3. Load SizeExtractR
     ```
     > library(SizeExtractR)
     ```

- **Save Path**
  Save a variable with the path string to the root directory folder containing the image analysis files. Do not use the '..', '~', or '\' symbols in the path directory.
  ```
  > mypath <- "<fill your directory>/Data_postImageJ_all"
  ```

- **Run Full_SizeExtractR_Workflow()**
  Note that the `known.calibration.length` parameter is entered as a value 10 (cm), as that was length of each calibration length on the measurement stick. Therefore, all computed size metrics will be in centimetres. The `include.calibrations` parameter is set to `FALSE` to avoid measurement stick lengths (e.g., *Cali_Pts* and *M1-M4*) being included in the final dataset.
  ```
  > data <- Full_SizeExtractR_Workflow(mypath,
                                       known.calibration.length = 10,
                                       include.calibrations = FALSE)
  ```

- **Step-by-step for `Full_SizeExtractR_Workflow()`**
  Everything this function does is described in the following steps, and will culminate in a full

calibrated size dataset. **However**, if mistakes are found during quality control, you will need to make changes outside R manually, and then rerun `Full_SizeExtractR_Workflow()`.
Note: For each following step there is a screen shot of the R console after running `Full_SizeExtractR_Workflow()`. **Please read the red text** which is a guide for navigating the interactive quality control checks and variable setting.

1. **Quality control - *Directory Variables***
   Ensures the folder names are all correct. If any names are incorrect, rename the folders manually outside of R, and rerun `Full_SizeExtractR_Workflow()`.

```
_______________________________________________________________________

N.B.
Please check that the following directory structure is correct.

    Each 'Directoy Level' should only show the folder names
(i.e., subdirectories) from that particular category, as defined
by the users own experimental design.

    For instance, for site surveys repeated over multiple years,
'Directoy Level 1' would show only years,
'Directoy Level 2' would show only Site IDs.

    If Directory Structure is wrong, or there are any mis-located
folders, then the folders must be re-organised into the correct
structure manually, and then the SizeExtractR tools can be used
. . . . . . . . . . . . . . . . . . . . . . . . . . . . . . . . .

__________________________

    Directory Level 1
. . . . . . . . . . . . . .

          Folder.Names
2016_during-bleaching
  2016_post-bleaching

__________________________

    Directory Level 2
. . . . . . . . . . . . . .

Folder.Names
       North
   Northwest

_____________________________________

Is the directory structure correct?
     (see above for details)
_____________________________________


1: Yes
2: No

Selection:
```

2. **Set Directory Variable names**
   The second step is to fill in the Variable names for each directory level. Here we have entered *Timepoint* and *Site*, which will end up as two categorical variables (columns) in the final dataset.

```
Good - directory is correct - continue.

______________________________________________

Enter a variable name for 'Directory Level 1'.
     (e.g. Year, no spaces allowed),
      see below: 1st few foldernames in this directory level
______________________________________________


          Folder.Names
 2016_during-bleaching
   2016_post-bleaching
Answer: Timepoint

______________________________________________

Enter a variable name for 'Directory Level 2'.
     (e.g. Year, no spaces allowed),
      see below: 1st few foldernames in this directory level
______________________________________________


 Folder.Names
        North
    Northwest
Answer: Site
```

3. **Quality control - ROI Type codes**
   Ensures there are no human errors (e.g., typos) in the annotated ROI Type codes (c.f. Figure 1). If errors are present choose no and then you will be given an option to locate the specific images that contain errors. Then you would need to rerun the workflow function. If there are no errors then proceed.

```
________________________________________________________________

 N.B.
 Please check that the ROI Type Names are correct.

 The Database formation function will not work if there are ny errors.

     ROI Type Names refer to the name given to that specific object
 during imageJ classification. 'M' and 'Cali_Pts' are the names given
 to the calibration points and measurement length object. These will be
 used for calibration. Other ROI Type Names are determined by the user.

. . . . . . . . . . . . . . . . . . . . . . . . . . . . . . . . .


__________________________

     ROI.Type
. . . . . . . . . . . . . .

 ROI.Type.Names
       Cali_Pts
              M
             Pa

_____________________________________

 Are the ROI Type Names Correct?
      (see above for details)

 _____________________________________


1: Yes
2: No

Selection: |
```

4. **Set ROI Variable names**
   Now we must link the user-defined ROI Label Codes to corresponding names of categorical ROI Variables for the final dataset. This information must be added manually to the **ROI_Labels.csv** template file outside the R environment. This template file will

have been automatically created. **<u>For this worked example fill the codes given in Table 4 into the .csv file manually, save it, then continue in R</u>** (see example in data repository). Please do this now.

```
Selection: 1

Good - ROI Types are correct - continue.
______________________________________________________________________

 .csv file 'ROI_Labels.csv' was just created in the path directory
 Follow the next 4 steps (outside of R):

    1) Open the .csv file
    2) Fill in the ROI label codes relavent to your image analysis
    3) Fill in the corresponding Variable names (no spaces)
    4) Save and close the .csv file
    5) Then once back in R choose 'Continue'

    NOTE: do not change the .csv filename or move to a different directory.
          see label characters from text file imports (may contain errors).

    NOTE: Do not include ROI label codes in the csv file if they have NOT
          been used any of the analysed images in your specified path.

    NOTE: Only include ROI Label Codes (alphabetical after the number in,
          the overall annotation - e.g. the 'bb' in 'Pa1bb'), and
          not the ROI Type codes (before the number - e.g., 'Pa').
 ______________________________________________________________________


 Label.Characters
                b
                p
                m


______________________________

 Is the new file 'ROI_Labels.csv'
 filled in correctly?

 ______________________________


1: Yes - Continue
2: No - Abort

Selection: |
```

5. **Quality control - ROI Label Codes 1**

   Ensures there are no human errors (e.g., typos) in the annotated *ROI Label Codes* (c.f. Fig. 4). Check that the data entered into the .csv file is correct.

```
____________________________________________________________________

 Double check that the ROI label codes correspond correctly to
 the Variable Names imported from the csv Template file

____________________________________________________________________


 ROI_Label_code Corresponding_Variable_Name
              b                        Pale
             bb                    Bleached
             pm            Partial_Mortality

_______________________________________

 Is the ROI labeling system correct?

 _______________________________________


1: Yes
2: No

Selection:
```

6. **Quality control - ROI Label Codes 2**

   Finally, check that the translation matrix from *ROI Label Codes* to *ROI Variables* is correct.

```
ROI.LabelCode  Pale Bleached Partial_Mortality
            b  TRUE    FALSE             FALSE
           bb FALSE     TRUE             FALSE
         bbpm FALSE     TRUE              TRUE
          bpm  TRUE    FALSE              TRUE
           pm FALSE    FALSE              TRUE
         <NA> FALSE    FALSE             FALSE

_______________________________________________

 Is the processed ROI labeling system correct?
         (see printed table above)
     Note: the ROI.label <NA> should be FALSE
           for all subsequent variables.

_______________________________________________


1: Yes
2: No

Selection: |
```

- **Congratulations**
  Now the database is calibrated. You have a single, quality-controlled, calibrated dataset of object sizes from the example image dataset. View the dataset using `View()` or `head()`. Notice the variable names we specified have been included in the dataset.

  The dataset can be saved using:
  `> write.csv(data, "Calibrated_Dataset.csv", row.names = FALSE)`

Table 5. Full dataset with all size metrics and user-defined categorical variables.

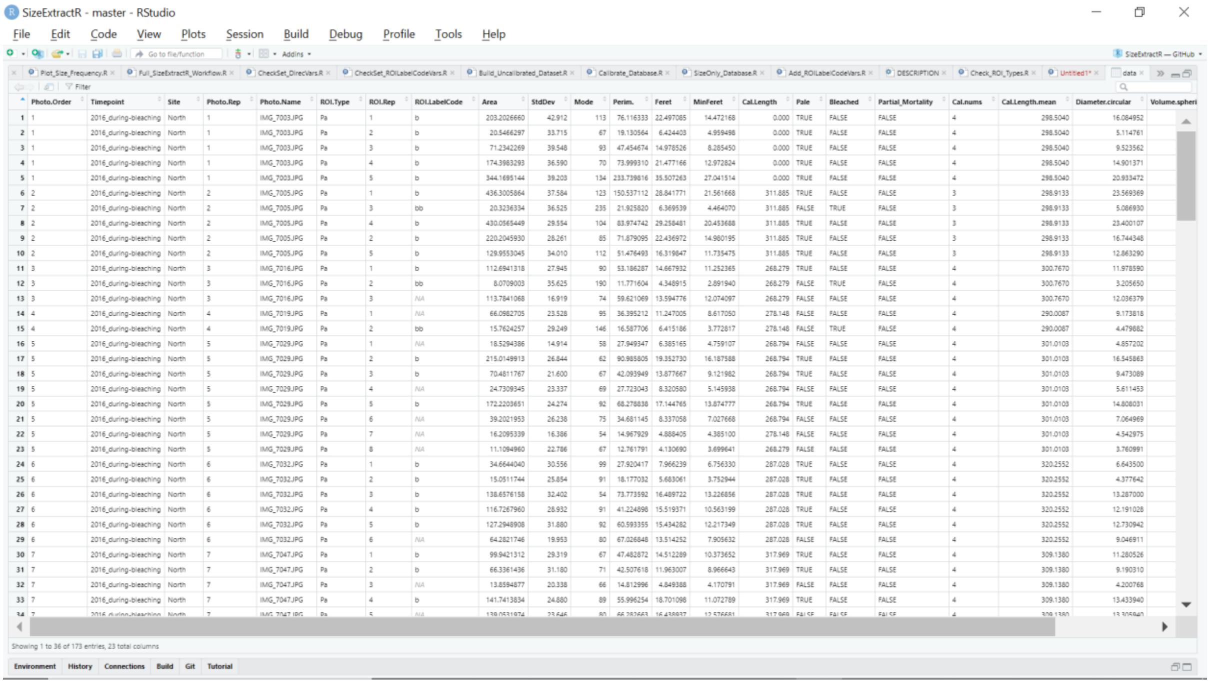

	Photo.Order	Timepoint	Site	Photo.Rep	Photo.Name	ROI.Type	ROI.Rep	ROI.LabelCode	Area	StdDev	Mode	Perim.	Feret	MinFeret	Cal.Length	Pale	Bleached	Partial_Mortality	Cal.nums	Cal.Length.mean	Diameter.circular	Volume.spheri
1	1	2016_during-bleaching	North	1	IMG_7003.JPG	Pa	1	b	203.2026660	42.912	113	76.116333	22.497085	14.472168	0.000	TRUE	FALSE	FALSE	4	298.5040	16.084952	
2	1	2016_during-bleaching	North	1	IMG_7003.JPG	Pa	2	b	20.5466297	33.715	67	19.130564	6.424403	4.959498	0.000	TRUE	FALSE	FALSE	4	298.5040	5.114761	
3	1	2016_during-bleaching	North	1	IMG_7003.JPG	Pa	3	b	71.2342269	39.548	93	47.454674	14.978526	8.285450	0.000	TRUE	FALSE	FALSE	4	298.5040	9.523562	
4	1	2016_during-bleaching	North	1	IMG_7003.JPG	Pa	4	b	174.3983293	36.590	70	73.999310	21.477166	12.972824	0.000	TRUE	FALSE	FALSE	4	298.5040	14.901371	
5	1	2016_during-bleaching	North	1	IMG_7003.JPG	Pa	5	b	344.1695144	39.203	134	233.739816	35.507263	27.041514	0.000	TRUE	FALSE	FALSE	4	298.5040	20.933472	
6	2	2016_during-bleaching	North	2	IMG_7005.JPG	Pa	1	b	436.3005864	37.584	123	150.537112	28.841771	21.561668	311.885	TRUE	FALSE	FALSE	3	298.9133	23.569369	
7	2	2016_during-bleaching	North	2	IMG_7005.JPG	Pa	3	bb	20.3236334	36.525	235	21.925820	6.369539	4.464070	311.885	FALSE	TRUE	FALSE	3	298.9133	5.086930	
8	2	2016_during-bleaching	North	2	IMG_7005.JPG	Pa	4	b	430.0565449	29.554	104	83.974742	29.258481	20.453688	311.885	TRUE	FALSE	FALSE	3	298.9133	23.400107	
9	2	2016_during-bleaching	North	2	IMG_7005.JPG	Pa	2	b	220.2045930	28.261	85	71.879095	22.436972	14.980195	311.885	TRUE	FALSE	FALSE	3	298.9133	16.744348	
10	2	2016_during-bleaching	North	2	IMG_7005.JPG	Pa	5	b	129.9553045	34.010	112	51.476493	16.319847	11.735475	311.885	TRUE	FALSE	FALSE	3	298.9133	12.863290	
11	3	2016_during-bleaching	North	3	IMG_7016.JPG	Pa	1	b	112.6941318	27.945	90	53.186287	14.667932	11.252365	268.279	TRUE	FALSE	FALSE	4	300.7670	11.978590	
12	3	2016_during-bleaching	North	3	IMG_7016.JPG	Pa	2	bb	8.0709003	35.625	190	11.771604	4.348915	2.891940	268.279	FALSE	TRUE	FALSE	4	300.7670	3.205650	
13	3	2016_during-bleaching	North	3	IMG_7016.JPG	Pa	3	NA	113.7841068	16.919	74	59.621069	13.594776	12.074097	268.279	FALSE	FALSE	FALSE	4	300.7670	12.036379	
14	4	2016_during-bleaching	North	4	IMG_7019.JPG	Pa	1	NA	66.0982705	23.528	95	36.395212	11.247005	8.617050	278.148	FALSE	FALSE	FALSE	4	290.0087	9.173818	
15	4	2016_during-bleaching	North	4	IMG_7019.JPG	Pa	2	bb	15.7624257	29.249	146	16.587706	6.415186	3.772817	278.148	FALSE	TRUE	FALSE	4	290.0087	4.479882	
16	5	2016_during-bleaching	North	5	IMG_7029.JPG	Pa	1	NA	18.5294386	14.914	58	27.949347	6.385165	4.759107	268.794	FALSE	FALSE	FALSE	4	301.0103	4.857202	
17	5	2016_during-bleaching	North	5	IMG_7029.JPG	Pa	2	b	215.0149913	26.844	62	90.985805	19.352730	16.187588	268.794	TRUE	FALSE	FALSE	4	301.0103	16.545863	
18	5	2016_during-bleaching	North	5	IMG_7029.JPG	Pa	3	b	70.4811767	21.600	67	42.093949	13.877667	9.121982	268.794	TRUE	FALSE	FALSE	4	301.0103	9.473089	
19	5	2016_during-bleaching	North	5	IMG_7029.JPG	Pa	4	NA	24.7309345	23.337	69	27.723043	8.320580	5.145938	268.794	FALSE	FALSE	FALSE	4	301.0103	5.611453	
20	5	2016_during-bleaching	North	5	IMG_7029.JPG	Pa	5	b	172.2203651	24.274	92	68.278838	17.144765	13.874777	268.794	TRUE	FALSE	FALSE	4	301.0103	14.808031	
21	5	2016_during-bleaching	North	5	IMG_7029.JPG	Pa	6	NA	39.2021953	26.238	75	34.681145	8.337058	7.027668	268.794	FALSE	FALSE	FALSE	4	301.0103	7.064969	
22	5	2016_during-bleaching	North	5	IMG_7029.JPG	Pa	7	NA	16.2095339	16.386	54	14.967929	4.888405	4.385100	278.148	FALSE	FALSE	FALSE	4	301.0103	4.542975	
23	5	2016_during-bleaching	North	5	IMG_7029.JPG	Pa	8	NA	11.1094960	22.786	67	12.761791	4.130690	3.699641	268.279	FALSE	FALSE	FALSE	4	301.0103	3.760991	
24	6	2016_during-bleaching	North	6	IMG_7032.JPG	Pa	1	b	34.6644040	30.556	99	27.920417	7.966239	6.756330	287.028	TRUE	FALSE	FALSE	4	320.2552	6.643500	
25	6	2016_during-bleaching	North	6	IMG_7032.JPG	Pa	2	b	15.0511744	25.854	91	18.177032	5.683061	3.752944	287.028	TRUE	FALSE	FALSE	4	320.2552	4.377642	
26	6	2016_during-bleaching	North	6	IMG_7032.JPG	Pa	3	b	138.6576158	32.402	54	73.773592	16.489722	13.226856	287.028	TRUE	FALSE	FALSE	4	320.2552	13.287000	
27	6	2016_during-bleaching	North	6	IMG_7032.JPG	Pa	4	b	116.7267960	28.932	91	41.224898	15.519371	10.563199	287.028	TRUE	FALSE	FALSE	4	320.2552	12.191028	
28	6	2016_during-bleaching	North	6	IMG_7032.JPG	Pa	5	b	127.2948908	31.880	92	60.593355	15.434282	12.217349	287.028	TRUE	FALSE	FALSE	4	320.2552	12.730942	
29	6	2016_during-bleaching	North	6	IMG_7032.JPG	Pa	6	NA	64.2821746	19.953	80	67.026848	13.514252	7.905632	287.028	FALSE	FALSE	FALSE	4	320.2552	9.046911	
30	7	2016_during-bleaching	North	7	IMG_7047.JPG	Pa	1	b	99.9421312	29.319	67	47.482872	14.512289	10.373652	317.969	TRUE	FALSE	FALSE	4	309.1380	11.280526	
31	7	2016_during-bleaching	North	7	IMG_7047.JPG	Pa	2	b	66.3361436	31.180	71	42.507618	11.963007	8.966643	317.969	TRUE	FALSE	FALSE	4	309.1380	9.190310	
32	7	2016_during-bleaching	North	7	IMG_7047.JPG	Pa	3	NA	13.8594877	20.338	66	14.812996	4.849388	4.170791	317.969	FALSE	FALSE	FALSE	4	309.1380	4.200768	
33	7	2016_during-bleaching	North	7	IMG_7047.JPG	Pa	4	b	141.7413834	24.880	89	55.996254	18.701098	11.072789	317.969	TRUE	FALSE	FALSE	4	309.1380	13.433940	
34	7	2016_during-bleaching	North	7	IMG_7047.JPG	Pa	5	NA	139.0531974	23.646	80	66.282663	16.438937	12.576681	317.969	FALSE	FALSE	FALSE	4	309.1380	13.305940	

Showing 1 to 36 of 173 entries, 23 total columns

Part 3.B.VI - Plotting size-frequency distributions in R.

Notice that the corals are more abundant in North Solitary Island than in Northwest Solitary island. Also see that there are fewer corals after the bleaching event across both islands.

```
> Plot_Size_Frequency(data,
                      size.metric = "Area",
                      log_size = TRUE,
                      nbins = 10,
                      group_by = c("Site", "Timepoint"),
                      facetRow_by = "Timepoint",
                      facetCol_by = "Site",
                      scales_gg = "fixed")
```

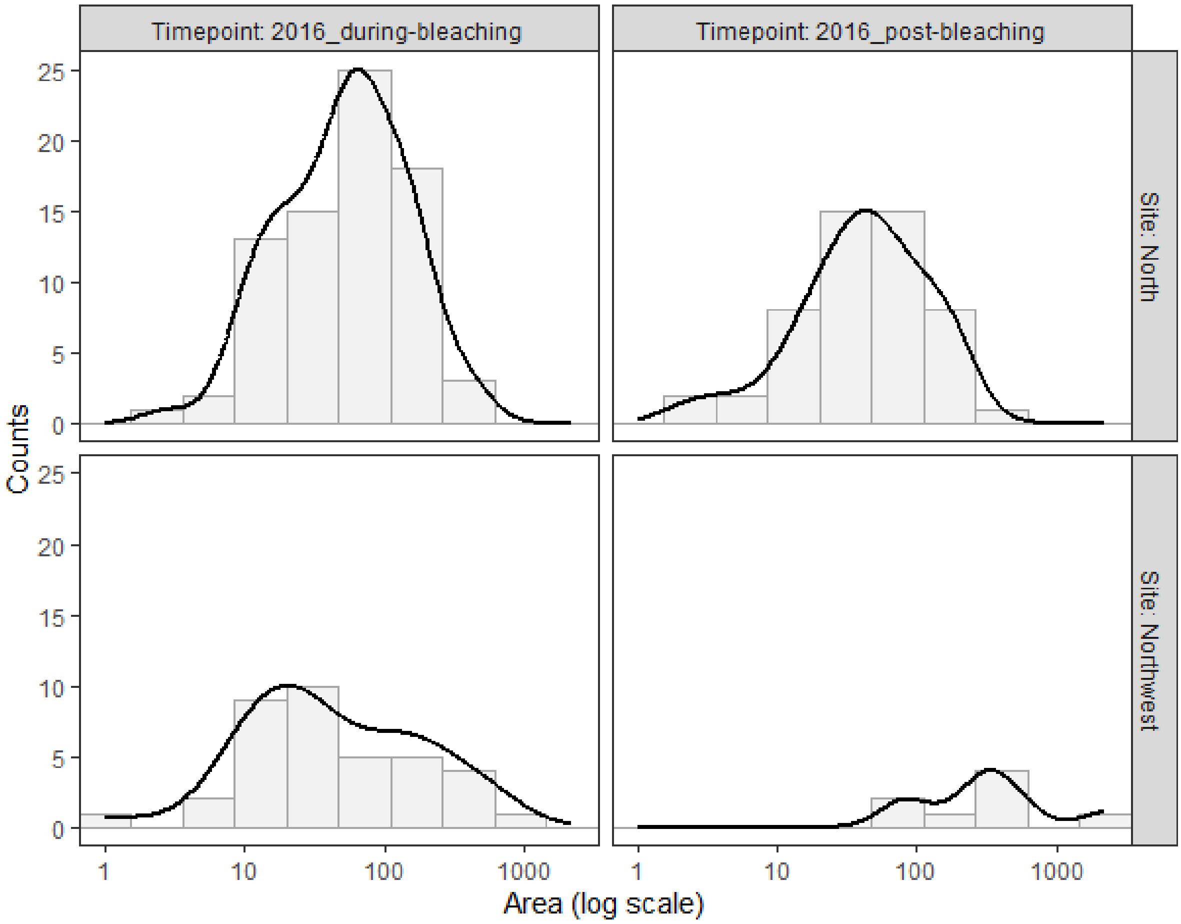

Part 3.C – Step-by-step without using the all-in-one- wrapper function

The Full SizeExtractR Workflow function is a wrapper for a series of other function. These can also be run individually, although we recommend running only the SizeExtractR workflow function instead.

The following lines of R code shows the exact order that the functions must be run using. Use this code, running line by line. Read the description in Table 2 below before running each function, so you understand what it does. Importantly, read the outputs given in the R terminal as it gives important instructions about whether your data is passing the data checks (see Part.3.B).

```
> varnames <- CheckSet_DirecVars(mypath)

> Database <- Build_Uncalibrated_Dataset(mypath, varnames)

> ROI_Types <- Check_ROI_Types(Database)

> Label.Translator <- CheckSet_ROILabelCodeVars(Database, mypath)

> Database.ROILabelCode <- Add_ROILabelCodeVars(Database,
Label.Translator)

> Database.cal <- Calibrate_Database(Database.ROILabelCode, known.length =
10)

> Database.SizeOnly <- SizeOnly_Database(Database.cal)
```

Table 2. Brief description of core R Functions. It is mandatory that Functions 1-7 are run in sequence. But note that a wrapper function runs this sequence automatically (see Table 3).

No.	Function / Inputs	Description
1	`CheckSet_DirecVars()`<br><br>Inputs<br>• path the directory path	Check and Set the Directory Variables (interactive).<br>• This function is used to check all the subfolder names in the source directory (Level zero) and their nested structure, to ensure it is in the right format to be translated into database variables (Part 2.B. Table 1).<br>• If this test fails, the user must manually edit the directory structure to get it to conform.<br>• Once the test passes the variable names are set by an interactive user input.<br>• Interactive function that will ask the user for feedback and inputs
2	`Build_Uncalibrated_Dataset()`<br><br>Inputs<br>• path the directory path<br>• `var.names` the output of Function 1	Build Uncalibrated Dataset (non-interactive).<br>• This function collates all data files from ImageJ analyses into a single uncalibrated dataset.<br>• Note that the ROI name labels are still not yet related to dataset variables.
3	`Check_ROI_Types()`<br><br>Inputs	Check ROI Types (interactive).<br>• This function checks the ROI Type codes.

	• `Database` the output of Function 2, an uncalibrated database	• As a reminder the ROI Type is the alphabetical code before the replicate number, that differentiates measurement lengths (i.e., *M*), calibration points (i.e., *Cali_Pts*), and user-defined objects of interest (e.g., *s* and *c*, for sponges and corals respectively).<br>• If there are issues in these codes or misspellings, the user will be prompted information about which images they are from and can then amend these in imageJ by opening/editing the specific image and ROIs zip file.<br>• Once issues are solved, and this check is passed you can move to the next check.
4	`CheckSet_ROILabelCodeVars()`<br><br>Inputs<br>• `Database` the output of Function 2, an uncalibrated database<br>• `path` the directory path	Check ROI Label Codes and set Variable names (interactive)<br>• This function checks the ROI Label Codes for any errors, and sets the variable names.<br>• As a reminder the ROI Label Code is the alphabetical code after the ROI Replicate number, that is fully user-defined. For example, the corals in the case study were given the codes "b" for bleached, or nothing for not bleached.<br>• This function creates a "label translator matrix" that is used by R to translate the ROI Label Codes into TRUE/FALSE database variables.<br>• The user must provide the labelling scheme in a csv file which is auto-created in the mypath directory folder.<br>• Once these checks are passed you can move on.<br>• If no ROI label codes are required, then user should still run the function, however, it will flag that no ROI Label Codes are used, and signal this to the next function.
5	`Add_ROILabelCodeVars()`<br><br>Inputs<br>• `Database` the output of Function 2, an uncalibrated database<br>• `label.translator` the output of Function 4, the "label translator matrix"	Add ROI Label Code Variables (non-interactive).<br>• This function adds the ROI Label Code Variables to the dataset via the "label translator matrix".<br>• The output is a new database that has all user-defined variables but is still uncalibrated.<br>• Once this runs you can move on.
6	`Calibrate_Database()`<br><br>Inputs	Calibrate Dataset (non-interactive).<br>• This function calibrates all the size metric variables based on the average number of pixels among the multiple calibration lengths (*M1-M4*) for each photo independently.

	• `Database.ROILabelCode` the output of Function 2 or Function 5, an uncalibrated database<br>• `known.length` the length of calibration measurement in users own units	• This is where the user final inputs the calibration length (in users own units, e.g., cm).<br>• The output is a full calibrated dataset, that still includes measurement lengths and calibration points (ROI Type = *M* and *Cali_Pts*).
7	`SizeOnly_Database()`<br><br>Inputs<br>• `Database.cal` the output of Function 6, a calibrated database	Size Only Dataset (non-interactive).<br>• This function removes all measurement lengths and calibration points (ROI Type = *M* and *Cali_Pts*) from the dataset.<br>• All remaining rows in the dataset are the ROIs of interest (i.e., corals and sponges) with calibrated size metrics and all user-defined variables.<br>• This can be saved and used for further analysis. See `write.csv()` for more details.

Part 3.D – Additional Functions (Full Workflow Function and Plotting Function)

As explained before, there are two additional functions in the SizeExtractR R-package that can be very useful. This are described briefly in Table 3, and are shown in more detail in the final two sections of this manual.

Full Workflow

The first is a Full Workflow function. This simply does the entire sequence of Table 2, except using only one function. Thus, this is an easier function to use, once the user understands what is happening under the hood.

Plotting

The second is a size distribution plotting function. You can plot size frequency distributions for any of the size metrics displayed as a histogram paired to a smoothed density curve. Size distributions can be compared between different grouping variables (up to three supported, e.g., Site, Year and Transect, Fig. 3). It does not matter how many categories are within each of these grouping variables. For full descriptions see the R help file.

Table 3. Brief description of addition R Functions. Function 8 is a wrapper function for all the core functions shown in Table 2, with the exact same usage. Function 9 is for plotting size distributions.

No.	Function / Inputs	Description
8	`Full_SizeExtractR_Workflow()`<br><br>Inputs<br>• `path` the directory path<br>• `known.calibration.length` the length of calibration measurements	Create Calibration Dataset in one function (interactive).<br>• This function is simply a wrapper of the 7 core sequentially-used function shown in Table 2.<br>• It performs all the same data checks, has the same variable setting use, and produces the same calibrated dataset.

	• `include.calibrations` a logical (TRUE/FALSE) that tells whether to include calibration lengths (*M* and *Cali_Pts*) or not in the final dataset.	• After a mistake is found in from one of the integrating checking functions, as you did before, simply amend it, and then rerun this `Full_SizeExtractR_Workflow()` function.
9	`Plot_Size_Frequency()`<br><br>Inputs<br>• `data` the output of Function 6, a calibrated database<br>• `size.metric` the size metric you wish to plot<br>• numerous other optional settings (see help file)	Plot Size Frequency Distribution plots (non-interactive).<br>• This function uses `ggplot2` to plot size-frequency distributions as a histogram and a smoothed probability density curve that is scaled to the histogram maximum height.<br>• Comparisons of size frequency distributions are made possible between multiple categorical variables (See Fig. 3).

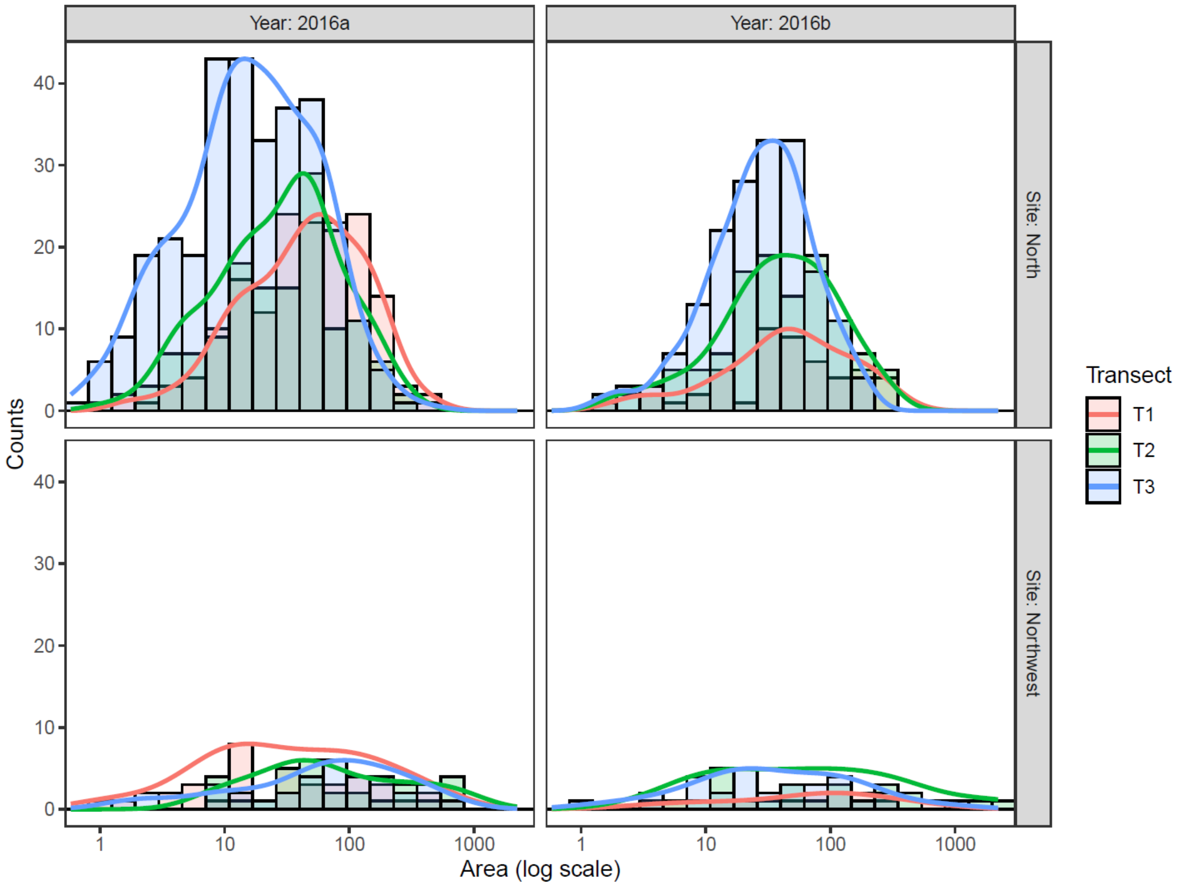

Figure 3. Example Size Frequency Distributions for *Pocillopora aliciae* in the Solitary Islands (Lachs et al. 2021) plotted using `Plot_Size_Frequency()`. The input dataset to this function was the SizeExtractR dataset. See code by reading the R documentation example in `?Plot_Size_Frequency()`, and change the code to suit your own dataset.
